# Association of variability in metabolic parameters with the incidence of type 2 diabetes: evidence from a functional community cohort

**DOI:** 10.1186/s12933-023-01922-4

**Published:** 2023-07-20

**Authors:** Ning Chen, Yu-Hong Liu, Li-Kun Hu, Lin-Lin Ma, Yu Zhang, Xi Chu, Jing Dong, Yu-Xiang Yan

**Affiliations:** 1 Department of Epidemiology and Biostatistics, Municipal Key Laboratory of Clinical Epidemiology, School of Public Health, Capital Medical University, Beijing, China; 2 Health Management Center, Xuanwu Hospital, Capital Medical University, Beijing, China

**Keywords:** Type 2 diabetes, Metabolic parameter, Variability, Cohort study

## Abstract

**Background:**

To investigate the association of variability in metabolic parameters such as total cholesterol concentrations (TC), uric acid (UA), body mass index (BMI), visceral adiposity index (VAI) and systolic blood pressure (SBP) with incident type 2 diabetes (T2D) and whether variability in these metabolic parameters has additive effects on the risk of T2D.

**Methods:**

Based on the Beijing Functional Community Cohort, 4392 participants who underwent three health examinations (2015, 2016, and 2017) were followed up for incident T2D until the end of 2021. Variability in metabolic parameters from three health examinations were assessed using the coefficient of variation, standard deviation, variability independent of the mean, and average real variability. High variability was defined as the highest quartile of variability index. Participants were grouped according to the number of high-variability metabolic parameters. Cox proportional hazards models were performed to assess the hazard ratio (HR) and 95% confidence interval (CI) for incident T2D.

**Results:**

During a median follow-up of 3.91 years, 249 cases of incident T2D were identified. High variability in TC, BMI, VAI and SBP was significantly associated with higher risks of incident T2D. As for UA, significant multiplicative interaction was found between variability in UA and variability in other four metabolic parameters for incident T2D. The risk of T2D significantly increased with the increasing numbers of high-variability metabolic parameters. Compared with the group with low variability for 5 parameters, the HR (95% CI) for participants with 1–2, 3, 4–5 high-variability metabolic parameters were 1.488 (1.051, 2.107), 2.036 (1.286, 3.222) and 3.017 (1.549, 5.877), respectively. Similar results were obtained in various sensitivity analyses.

**Conclusions:**

High variability of TC, BMI, VAI and SBP were independent predictors of incident T2D, respectively. There was a graded association between the number of high-variability metabolic parameters and incident T2D.

**Supplementary Information:**

The online version contains supplementary material available at 10.1186/s12933-023-01922-4.

## Background

Type 2 diabetes (T2D) accounts for around 90% of diabetes worldwide and has become a serious public health problem [1]. As a common metabolic disease, the significant association between the development of T2D and various metabolic indicators such as blood pressure (BP), lipids, obesity or abdominal obesity, and uric acid (UA) have been generally confirmed [2–4]. However, most prior studies were based on a single baseline metabolic parameter measurement, which may not reflect long-term exposure. Therefore, it is essential to identify whether variability in metabolic parameters is associated with incident T2D. Moreover, such studies may provide further evidence for future interventional trials that focus on reducing metabolic parameters variability in addition to average metabolic parameters levels.

Studies have confirmed that visit-to-visit variability in metabolic parameters could predict the risk of several adverse outcomes and mortality, independent of baseline and average levels. Two Chinese studies found the significant association between incident cardiovascular disease (CVD) and triglyceride-glucose index (TyG) variability, an index to simply assess insulin resistance [5, 6]. A pooled cohort analysis conducted in Iran found that changes in fasting plasma glucose (FBG) status were significantly associated with the risk of mortality in individuals without diabetes [7]. Based on nationally representative data from the Korean National Health Insurance System, Mee Kyoung Kim et al. confirmed the significant association of the number of high variability in BP, FBG, cholesterol concentrations and body mass index (BMI) with mortality and cardiovascular outcomes [8]. As for T2D, limited studies have examined repeated metabolic parameters measurements to evaluate the impact of longitudinal metabolic parameters variability on the risk of incident T2D in China. Besides, few evidence confirmed the association between the number of high-variability metabolic parameters and incident T2D.

Therefore, based on the Beijing functional community cohort, we aimed to investigate the relationship between variability in several visit-to-visit metabolic parameters, including total cholesterol concentrations (TC), UA, BMI, visceral adiposity index (VAI) and systolic blood pressure (SBP), with incident T2D and further evaluate whether they have additive effects on the risk of T2D.

## Methods

### Study design and subjects

The present study was performed based on a prospective cohort design and the aim was to evaluate the association of variability in visit-to-visit metabolic parameters with incident T2D. Participants who underwent annual health examination from 2015 to 2017 (baseline and index year) were included and variability in several metabolic parameters from three health examination were calculated (TC, UA, BMI, VAI and SBP) as predictors of future T2D. All the participants were annually followed under same identical conditions for the development of T2D until December 31, 2021.

The population data used in this study were obtained from the Beijing Functional Community Cohort, which was initiated in 2010 and 8671 individuals with annual examination were recruited at the Health Management Center of Xuanwu Hospital, Capital Medical University [9]. These participants were aged 30 ~ 65 years and from various occupations in Xicheng District, Beijing, including medical workers, teaching staff, government workers, workers, and service industries, representing most of the occupational population in Beijing. The inclusion and exclusion criteria of the participants were described previously [9]. The study protocol was approved by the ethics committee of Capital Medical University and Xuanwu Hospital, and it was conducted according to the principles of the Declaration of Helsinki. All study subjects were informed at enrollment.

**Fig. 1:**
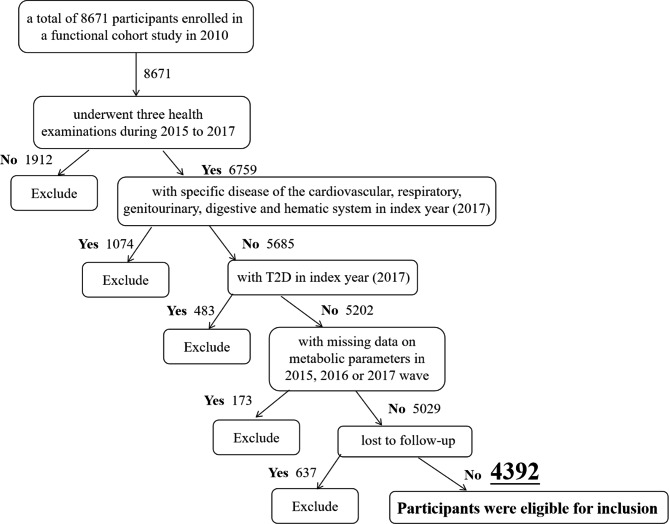
Flow chart of this study

In this analysis, we included participants who underwent three health examinations between January 2015 and December 2017 (baseline and index year). Of 6759 participants, we excluded those with T2D in 2017 (n = 483), those with missing data on metabolic parameters in 2015 to 2017 wave (n = 173) and those who were lost to follow-up (n = 637). We also excluded individuals who met the diagnostic criteria of specific disease of cardiovascular system, respiratory system, genitourinary system, digestive system and hematic system according to the medical records in 2017. Finally, 4392 participants aged 37 ~ 72 years old in 2017 were included in our analysis and were annually followed-up for the development of T2D until December 31, 2021 (Fig. 1).

### Data collection and clinical measurements

Structured standard questionnaires were used to collect basic information about the study participants, including sex, age, smoking status, alcohol intake, education level, physical activity, disease history, family history of disease and medication use (such as hypoglycemic agents, antihypertensive agents and lipid-lowering agents). Smoking status was categorized into three groups: non-smoker (never smoked), ex-smoker (quit smoking) and current smoker (smoked ≥ 1 cigarette per day). Alcohol intake was categorized into three groups: no alcohol intake, low risk alcohol intake (intake of wine/beer/cider/spirits < 1 time per month) and high risk alcohol intake (intake of wine/beer/cider/spirits ≥ 1 time per month). Physical activity was defined as walking or cycling ≥ 15 minutes per day, exercising or physical activity > 2 hours per week or lifting or carrying heavy loads at work per day. Family history of diabetes (FHD) was defined as having diabetes in first-degree relatives.

Height and weight were measured while the participants wearing light clothing without shoes, and BMI was calculated as weight in kilograms divided by the square of height in meters (kg/m^2^). Waist circumstance (WC) was measured at the height of the navel upon breath intake using a non-extendable linen measure. Blood pressure (BP) was averaged after three consecutive measurements using a standard mercury sphygmomanometer on the right arm of the study subject after 5 minutes of sitting still.

Venous blood samples were collected between 7:30 and 8:30 am after an overnight fast. In a calm state, all samples were immediately centrifuged for laboratory measurements. TC, high-density lipoprotein cholesterol (HDL-C), low-density lipoprotein cholesterol (LDL-C), triglycerides (TG), aspartate aminotransferase (AST), alanine aminotransferase (ALT), creatinine (Cr), Urea and UA were measured using standard laboratory methods (Hitachi Autoanalyzer 7060; Hitachi, Tokyo, Japan). Fasting blood glucose (FBG) levels were determined by the glucose oxidase method. Hemoglobin A1c (HbA1c) levels were measured by Ion-exchange chromatography.

In this study, VAI was used to assess visceral adiposity of the participants. The sex specific formula of VAI is as follows [10]:

Men: VAI = WC/(39.68 + 1.88*BMI)*(TG/1.03)*(1.31/HDL-C)

Women: VAI = WC/(36.58 + 1.89*BMI)*(TG/0.813)*(1.52/HDL-C)

### Definition of Parameter Variability

Variability was defined as intraindividual variability in TC, UA, BMI, VAI and SBP values measured in 2015, 2016, and 2017. Four indices of variability were used: (1) coefficient of variation (CV), (2) standard deviation (SD), (3) variability independent of the mean (VIM) and (4) average real variability (ARV) [8, 11]. Taking TC as an example, the VIM is calculated first as the SD of TC divided by the mean TC raised to the power of *x*, where *x* is obtained from fitting a nonlinear regression model among the entire sample where SD = a*mean^*x*^. This quantity is then multiplied by the sample mean TC raised to the power of *x*. As such,

$$\begin{array}{l}VIM = \frac{{k*SD(TC)}}{{Mean{{(TC)}^x}}}\\Where,k = Mean{(Mean(TC))^x}\end{array}$$

ARV is the average of the absolute differences between consecutive values and was calculated using the following formula, where N denotes the number of measurements of the metabolic parameters. As such,

$$\eqalign{ARV = \frac{1}{{N - 1}}\sum\limits_{k = 1}^{N - 1} & {\left| {Valu{e_{k + 1}} - Valu{e_k}} \right|}, \cr & {\rm{ in\, this\, study}},{\rm{ N}} = 3.}$$

High variability was defined as the highest quartile (Q4) of variability index, and low variability was defined as the lower 3 quartiles (Q1–Q3) of variability index. According to the number of high-variability metabolic parameters (TC, UA, BMI, VAI and SBP), the participants were classified into Group 1 (with 0 high-variability metabolic parameters), Group 2 (with 1 and 2 high-variability metabolic parameters), Group 3 (with 3 high-variability metabolic parameters), and Group 4 (with 4 and 5 high-variability metabolic parameters).

### Definition of type 2 diabetes

According to Chinese Guidelines for the Prevention and Treatment of Type 2 Diabetes (2013 Edition), the diagnosis of T2D in China used the classification criteria for glucose metabolic status and the diagnostic criteria for T2D proposed by WHO in 1999 [12]. T2D was defined as: 1) FBG ≥ 7.0 mmol/L, 2) random plasma glucose ≥ 11.1mmol/L, 3) 2-h plasma glucose in oral glucose tolerance test (OGTT) ≥ 11.1mmol/L. In 2011, WHO recommended HbA1c ≥ 6.5% as a diagnostic criterion for T2D. In this study, those who met any of the above criteria can be diagnosed as T2D.

### Statistical analysis

Categorical variables were presented as number (proportions), continuous variables were presented as mean ± SD or median (quartiles). Differences between groups were compared using Chi-square test for categorical variables and One-way ANOVA or Kruskal-Wallis rank sum test for continuous variables, as appropriate.

In this study, CV was firstly used to determine the variability of five parameters. Spearman’s correlation was conducted to describe the relationships between variability in TC, UA, BMI, VAI and SBP. During the follow-up period, the incidence rates of T2D for each group were calculated. Time to first T2D event was examined using Kaplan-Meier survival curves and compared using Log-rank test. Multivariable-adjusted Cox proportional hazards regression models were used to estimate the adjusted hazard ratios (HR) and 95% confidence intervals (CI). Model 1 was adjusted for baseline age, sex, smoking status, alcohol intake, education and physical activity. Model 2 was further adjusted for baseline WC, SBP, diastolic blood pressure (DBP), BMI, VAI, TC, LDL-C, HDL-C, TG, ALT, AST, Cr, Urea, UA, FBG, HbA1c, FHD, medication use (antihypertensive agents and lipid-lowering agents) based on model 1. C statistics, net reclassification improvement (NRI) and integrated discrimination improvement (IDI) were used to estimate the improvement in discrimination and reclassification after adding the variability of these metabolic parameters to the clinical risk model of incident T2D prediction.

We performed multiple sensitivity analyses. First, SD, VIM and ARV were used to determine the variability of parameters. Second, participants experiencing T2D within one year were excluded. Third, the mean values of these metabolic parameters from 2015 to 2017 instead of baseline values were adjusted in Model 2 of the Cox proportional-hazards model. The potential effect modification by sex, age and BMI categories was evaluated using stratified analysis and interaction testing using a likelihood ratio test.

Statistical analyses were performed using R software (version: 4.2.1; R Foundation for Statistical Computing) and MedCalc® Statistical Software (version 20.100; MedCalc Software Ltd, Ostend, Belgium; https://www.medcalc.org; 2022). The difference was considered statistically significant at two-side significance level of *P* < 0.05.

## Results

The final analysis included 4392 individuals, and 2008 (45.72%) were men. The mean (SD) age of these participants was 52.86 (9.69) years. Table 1 presented the baseline characteristics of the study participants. As Table 1 listed, participants with more high-variability parameters were more likely to be women. Participants in Group 4 had the highest baseline VAI value, while there were no significant differences of baseline TC, UA, BMI or SBP among different groups. The CV of each parameter increased gradually with the number of high-variability parameters (*P <* 0.001).

**Table 1:** Baseline characteristics of study population in 2017

	Total	Group 1	Group 2	Group 3	Group 4	*P*
N	4392	1149	2726	425	92	
Age, y	52.86 ± 9.69	52.98 ± 9.79	52.96 ± 9.64	51.93 ± 9.62	52.38 ± 10.06	0.202
Men, n (%)	2008 (45.72)	593 (41.79)	1215 (44.57)	165 (38.82)	35 (38.04)	<0.001
Smoking, n (%)						0.704
Non-smoker	3972 (90.44)	1031 (89.73)	2471 (90.55)	387 (91.06)	83 (90.22)	
Ex-smoker	157 (3.57)	41 (3.57)	96 (3.52)	18 (4.24)	2 (2.17)	
Current smoker	263 (5.99)	77 (6.70)	159 (5.83)	20 (4.71)	7 (7.61)	
Drinking, n (%)						0.110
No alcohol intake	3733 (85.00)	968 (84.25)	2331 (85.51)	354 (83.29)	80 (86.96)	
Low risk alcohol intake	149 (3.39)	29 (2.52)	95 (3.48)	21 (4.94)	4 (4.35)	
High risk alcohol intake	510 (11.61)	152 (13.23)	300 (11.01)	50 (11.76)	8 (8.70)	
Physical activity, n (%)	3209 (73.06)	843 (59.41)	2004 (73.51)	293 (68.94)	69 (75.00)	0.243
Education, n (%)						0.893
Primary school and below	120 (2.73)	30 (2.11)	79 (2.90)	9 (2.12)	2 (2.17)	
Junior high school	1162 (26.46)	292 (20.58)	727 (26.67)	119 (28.00)	24 (26.09)	
High school and above	3110 (70.81)	827 (58.28)	1920 (70.43)	297 (69.88)	66 (71.74)	
WC, cm	82 [75, 89]	83 [76, 90]	82 [75, 89]	81 [75, 88]	81 [75, 91]	0.060
Medication use, n (%)						
Lipid-lowering agents	116 (2.64)	35 (3.05)	53 (1.94)	20 (4.71)	8 (8.70)	<0.001
Antihypertensive agents	404 (9.20)	103 (8.96)	218 (8.00)	69 (16.24)	14 (15.22)	<0.001
FHD, n (%)	844 (19.22)	235 (20.45)	504 (18.49)	83 (19.53)	22 (23.91)	0.335
DBP, mmHg	76 [69, 83]	77 [70, 84]	75 [68, 83]	74 [67, 81]	75 [70, 82]	<0.001
LDL-C, mmol/L	2.93 [2.43, 3.49]	3.00 [2.52, 3.52]	2.92 [2.41, 3.48]	2.80 [2.27, 3.44]	2.94 [2.19, 3.58]	<0.001
HDL-C, mmol/L	1.40 [1.18, 1.66]	1.41 [1.18, 1.66]	1.41 [1.18, 1.67]	1.39 [1.15, 1.65]	1.33 [1.08, 1.63]	0.410
TG, mmol/L	1.31 [0.92, 1.87]	1.29 [0.92, 1.76]	1.30 [0.92, 1.88]	1.40 [0.96, 2.11]	1.52 [0.93, 2.53]	0.001
AST, U/L	21 [18, 25]	21 [18, 24]	21 [18, 25]	22 [18, 26]	22 18, 27]	0.005
ALT, U/L	19 [14, 26]	19 [14, 26]	19 [14, 26]	19 [15, 26]	19 [14, 28]	0.706
FBG, mmol/L	5.07 [4.77, 5.40]	5.05 [4.75, 5.39]	5.07 [4.77, 5.39]	5.10 [4.78, 5.43]	5.18 [4.87, 5.58]	0.033
HbA1c, %	5.4 [5.0, 5.6]	5.4 [5.0, 5.6]	5.4 [5.1, 5.6]	5.3 [5.0, 5.6]	5.4 [5.2, 5.6]	0.263
Cr, µmol/L	64 [55, 75]	66 [57, 76]	64 [55, 75]	63 [53, 74]	62 [53, 74]	0.001
Urea, mmol/L	4.91 [4.16, 5.82]	4.96 [4.18, 5.93]	4.90 [4.17, 5.79]	4.86 [4.09, 5.74]	4.86 [3.93, 5.82]	0.326
TC, mmol/L	4.76 [4.23, 5.36]	4.79 [4.30, 5.36]	4.75 [4.21, 5.35]	4.66 [4.13, 5.35]	4.85 [4.18, 5.52]	0.092
UA, µmol/L	316 [264, 377]	323 [272, 385]	313 [264, 374]	311 [254, 373]	323 [245.75, 383.75]	0.053
BMI, kg/m^2^	24.39 [22.19, 26.71]	22.23 [24.54, 26.89]	24.33 [22.15, 26.58]	24.09 [22.21, 26.77]	25.06 [22.04, 27.76]	0.405
VAI	1.41 [0.91, 2.29]	1.33 [0.89, 2.11]	1.41 [0.91, 2.29]	1.57 [0.96, 2.80]	1.82 [0.97, 3.46]	<0.001
SBP, mmHg	122 [112, 135]	124 [113, 135]	122 [112, 134]	124 [111, 139]	127 [115, 135.25]	0.027
Variability, %						
TC	6.52 [4.12, 9.83]	5.17 [3.40, 7.05]	6.82 [4.25, 10.23]	10.94 [7.22, 15.17]	12.62 [10.55, 17.26]	<0.001
UA	9.95 [6.29, 14.45]	8.03 [5.21, 10.91]	10.33 [6.37, 15.12]	15.52 [9.54, 19.01]	19.40 [15.88, 23.60]	<0.001
BMI	2.42 [1.53, 3.45]	1.97 [1.32, 2.64]	2.50 [1.57, 3.61]	3.66 [2.35, 4.75]	4.12 [3.51, 4.98]	<0.001
VAI	28.42 [18.36, 39.85]	23.55 [15.77, 30.66]	29.28 [18.67, 41.44]	43.35 [25.77, 52.63]	49.25 [43.18, 61.27]	<0.001
SBP	5.68 [3.68, 8.20]	4.69 [93.06, 6.24]	5.85 [3.76, 8.63]	8.49 [5.38, 10.41]	9.56 [8.42, 11.81]	<0.001

WC: waist circumstance, FHD: Family history of diabetesDBP: diastolic blood pressure, LDL-C: low-density lipoprotein cholesterol, HDL-C: high-density lipoprotein cholesterol, TG: triglycerides, AST: aspartate aminotransferase, ALT: alanine aminotransferase, FBG: fasting blood glucose, HbA1c: hemoglobin A1c, Cr: creatinine, TC: total cholesterol, BMI: body mass index, UA: uric acid, VAI: visceral adiposity index, SBP: systolic blood pressure

The correlation between different metabolic parameters variability were shown in Fig S1 in Additional file 1. The correlation between TC variability and UA variability, BMI variability, VAI variability, SBP variability were all significant but not robust (*P <* 0.05, r < 0.10). Another significant correlation was observed between BMI variability and VAI variability, but this correlation was also not robust (*P <* 0.05, r = 0.059).

**Fig. 2:**
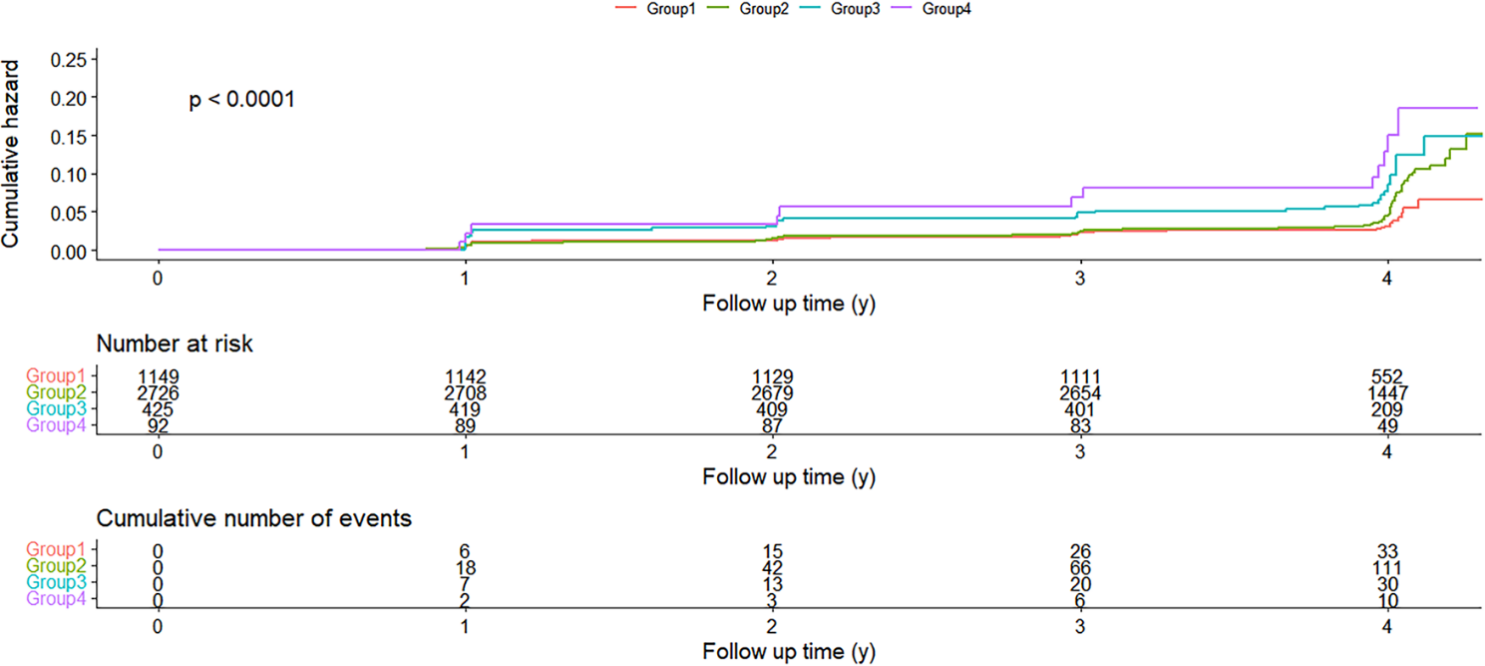
Kaplan-Meier curves of the incidence of type 2 diabetes according to different groups

**Fig. 3:**
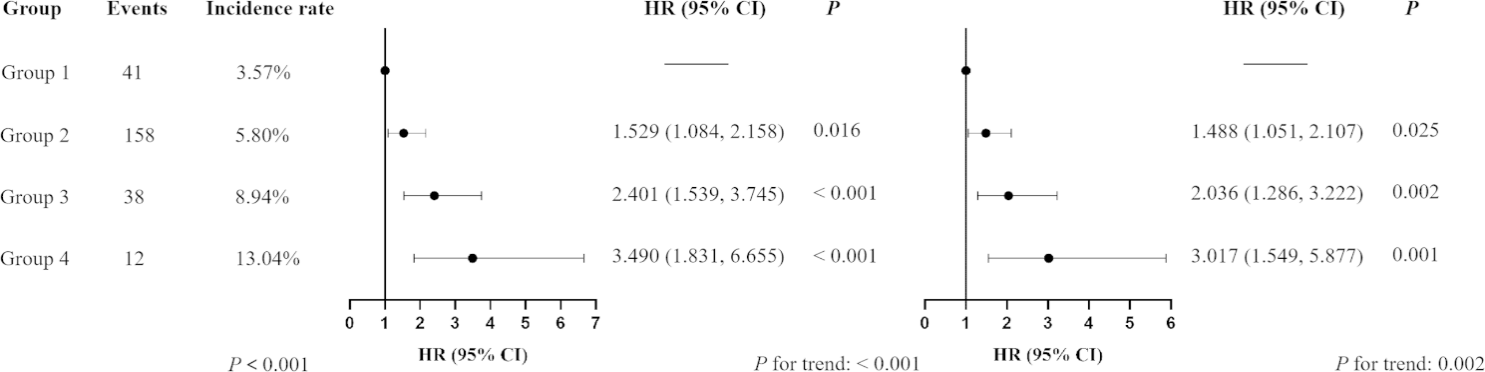
Association between the number of high-variability metabolic parameters and the incidence of type 2 diabetes

After a median follow-up of 3.91 years, 249 cases of incident T2D were identified (155 participants with FBG ≥ 7.0 mmol/L and HbA1c ≥ 6.5%, 49 participants with FBG ≥ 7.0 mmol/L, 45 participants with HbA1c ≥ 6.5%). As was shown in Fig. 2, the Kaplan-Meier curves showed that participants with more high-variability parameters had higher risk of incident T2D than those with low variability of all 5 parameters (Log-rank test, *P <* 0.0001). Participants with more high-variability metabolic parameters had a higher prevalence of T2D (*P <* 0.001) and T2D incidence among participants with 0, 1–2, 3, 4–5 high-variability metabolic parameters were 3.57%, 5.80%, 8.94% and 13.04%, respectively (Fig. 3).

As Fig. 3 presented, after adjusting for potential confounders, compared with the group with low variability of all 5 parameters (reference group), the HRs and 95% CI for participants in Group 2, Group 3 and Group 4 were 1.488 (95% CI: 1.051, 2.107), 2.036 (95% CI: 1.286, 3.222) and 3.017 (95% CI: 1.549, 5.877), respectively. As was shown in Table S1 in Additional file 1, when we re-run the analysis based on the number of high-variability metabolic parameters (0–5), we obtained the same results and participants with 5 high-variability metabolic parameters had a significantly higher prevalence (27.27%) and risk of incident T2D (HR: 4.473, 95% CI: 1.281, 15.615) than other group. The risk of T2D significantly increased with the number of high variability parameters (*P* for trend < 0.001).

When the variability index was used as a continuous variable, increase in variability of TC, BMI, VAI and SBP were significantly associated with increased risk of T2D after full multi-variable adjustment (Additional file 1: Table S2). For each 5% increase in CV of TC, BMI, VAI and SBP, the risk of incident T2D increased by 15.3% (HR: 1.153, 95% CI: 1.054, 1.260), 74.8% (HR: 1.748, 95% CI: 1.777, 2.597), 4.0% (HR: 1.040, 95% CI: 1.004, 1.077) and 16.7% (HR: 1.167, 95% CI: 1.019, 1.337). As for UA, the association between increase in its variability and incident T2D was not significant. Considering the aforementioned finding that participants with five high-variability parameters had a significantly higher incidence and risk of developing T2D, we further performed a test for interaction effects and significant interaction between the 5% increase in CV of UA and 5% increase in CV of TC, BMI, VAI or SBP were found for incident T2D. As was shown in Table 2, for CV quartiles of each metabolic parameter, an incrementally higher risk of the incidence of T2D for higher CV quartiles of TC, BMI, VAI and SBP compared with the lowest quartile group were observed (*P* for trend < 0.05). The association between variability in each parameter and the incidence of T2D were confirmed after adjusting for baseline TC, UA, BMI, VAI and SBP. We further assessed the association of variability in some other parameters with incident T2D and found that variability of DBP and LDL-C could also significantly increase the risk of T2D (Table S3 in Additional file 1).

As was shown in Table 3, C-statistic, NRI and IDI were used to calculate the incremental predictive value of the variability in metabolic parameters for the incidence of T2D and so as to evaluate the influence of the variability in metabolic parameters. The addition of variability in different metabolic parameters to the clinical risk model for incident T2D prediction all increased the C-statistic and continuous NRI. Then we added different numbers of metabolic parameters variability into same model and found that the addition of variability in TC, UA, BMI and SBP showed the best discrimination and reclassification improvement. After adding the variability of TC, UA, BMI and SBP to the clinical risk model, the C-statistic significantly increased from 0.765 (95% CI: 0.752, 0.777) to 0.785 (95% CI: 0.772, 0.797) (difference: 0.020; *P* < 0.001); and there was a significant reclassification improvement (continuous NRI: 29.33%, 95% CI: 16.58%, 42.08%, *P <* 0.001; IDI: 0.71%, 95% CI: 0.14%, 1.28%, *P* = 0.015).

The results were similar when the variability of parameters were determined by SD, VIM and ARV (Additional file 1: Table S4-S6). The number of high-variability metabolic parameters as measured by SD, VIM or ARV were all independent predictors of the incidence of T2D after multi-variable adjustment. Then we repeated the analysis after excluding participants who developed T2D within one year and we still observed incrementally higher incidence rates and risk of T2D with an increasing number of high-variability metabolic parameters. Finally, the mean levels of TC, UA, BMI, VAI and SBP were adjusted instead of the baseline levels in the Cox proportional-hazards model and nearly identical results were found.

**Fig. 4:**
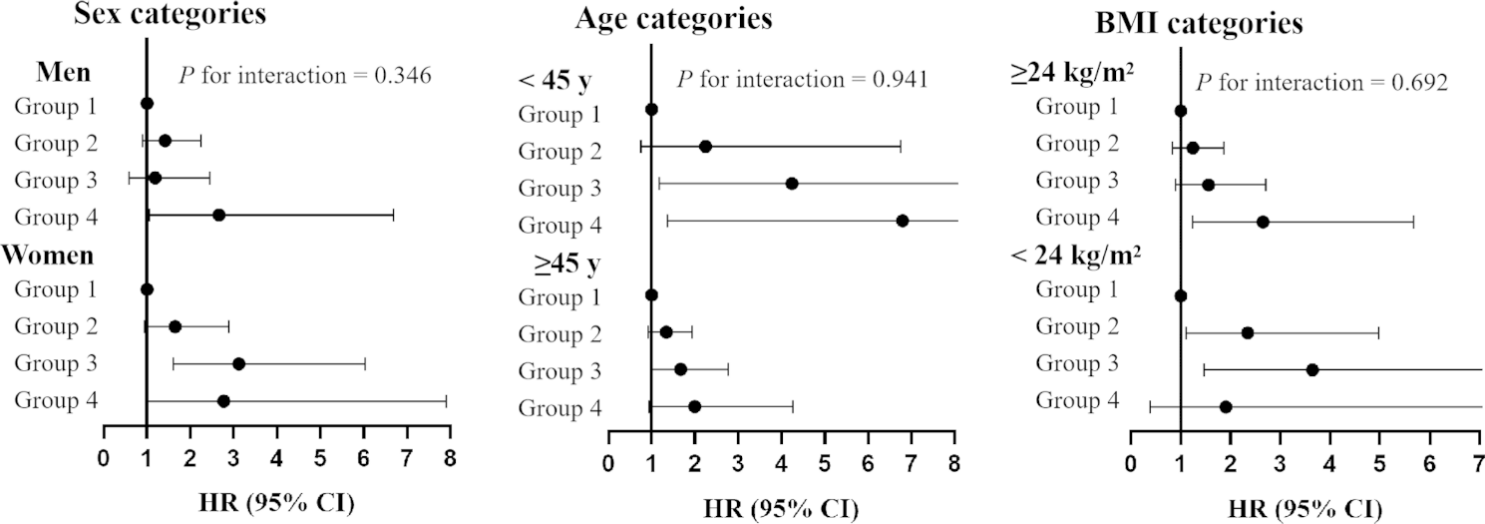
Subgroup analyses: association between the number of high-variability metabolic parameters and the incidence of type 2 diabetes

Subgroup analyses stratified by sex, age and BMI were performed and we did not find significant interaction between the number of high-variability parameters and sex (men, women), age (< 45 years, ≥ 45 years), or BMI (< 24 kg/m^2^, ≥ 24 kg/m^2^) (Fig. 4).

**Table 2:** Association between quartiles of variability of metabolic parameters and the incidence of T2D

	Model 1 ^a^		Model 2 ^b^	
	HR (95% CI)	*P*	HR (95% CI)	*P*
**TC**				
Q1				
Q2	1.277 (0.840, 1.943)	0.252	1.283 (0.842, 1.956)	0.246
Q3	1.772 (1.195, 2.628)	0.004	1.859 (1.252, 2.762)	0.002
Q4	2.209 (1.517, 3.219)	< 0.001	1.984 (1.357, 2.901)	< 0.001
*P* for trend		< 0.001		0.001
**UA**				
Q1				
Q2	0.985 (0.682, 1.423)	0.937	0.958 (0.661, 1.389)	0.822
Q3	1.070 (0.748, 1.532)	0.710	1.144 (0.798, 1.641)	0.464
Q4	1.293 (0.908, 1.840)	0.154	1.224 (0.856, 1.748)	0.268
*P* for trend		0.394		0.511
**BMI**				
Q1				
Q2	0.993 (0.682, 1.447)	0.972	0.948 (0.648, 1.385)	0.781
Q3	1.123 (0.775, 1.629)	0.539	1.202 (0.827, 1.749)	0.335
Q4	1.660 (1.179, 2.336)	0.004	1.667 (1.179, 2.358)	0.004
*P* for trend		0.005		0.004
**VAI**				
Q1				
Q2	1.142 (0.762, 1.710)	0.520	1.125 (0.748, 1.692)	0.571
Q3	1.736 (1.197, 2.517)	0.004	1.624 (1.117, 2.361)	0.011
Q4	1.894 (1.308, 2.744)	0.001	1.593 (1.083, 2.343)	0.018
*P* for trend		0.001		0.019
**SBP**				
Q1				
Q2	1.575 (1.041, 2.382)	0.032	1.713 (1.130, 2.597)	0.011
Q3	1.829 (1.234, 2.741)	0.003	1.986 (1.329, 2.968)	0.001
Q4	2.008 (1.362, 2.961)	< 0.001	1.973 (1.333, 2.920)	0.001
*P* for trend		0.004		0.004

TC: total cholesterol, BMI: body mass index, UA: uric acid, VAI: visceral adiposity index, SBP: systolic blood pressure, T2D: type 2 diabetes

^a^ Model 1 was adjusted for age, sex, smoking status, alcohol intake, education and physical activity.

^b^ Model 2 was adjusted for age, sex, smoking status, alcohol intake, education and physical activity, waist circumstance, systolic blood pressure, diastolic blood pressure, body mass index, visceral adiposity index, total cholesterol, low-density lipoprotein cholesterol, high-density lipoprotein cholesterol, triglycerides, aspartate aminotransferase, alanine aminotransferase, creatinine, Urea, uric acid, fasting blood glucose, hemoglobin A1c, family history of diabetes, medication use (antihypertensive agents and lipid-lowering agents)

**Table 3:** Reclassifcation and discrimination statistics for predicting T2D by adding variability of metabolic parameters

	C statistics (95% CI)	*P*	Continuous NRI (95% CI), %	*P*	IDI (95% CI), %	*P*
Clinical model ^a^	0.765 (0.752, 0.777)		-		-	
+TC	0.774 (0.762, 0.787)	**0.003**	13.33 (0.65, 26.01)	**0.039**	0.13 (-0.23, 0.49)	0.483
+UA	0.770 (0.757, 0.782)	**0.011**	13.07 (0.30, 25.84)	**0.045**	0.03 (-0.20, 0.26)	0.795
+BMI	0.770 (0.757, 0.782)	0.091	20.41 (7.64, 33.18)	**0.002**	0.35 (0.03, 0.68)	**0.035**
+VAI	0.768 (0.756, 0.781)	0.104	14.67 (1.88, 27.45)	**0.025**	0.15 (-0.09, 0.39)	0.227
+SBP	0.769 (0.756, 0.781)	**0.026**	14.22 (1.45, 27.00)	**0.029**	0.20 (-0.03, 0.43)	0.087
+TC, UA, BMI, SBP	0.785 (0.772, 0.797)	**< 0.001**	29.33 (16.58, 42.08)	**< 0.001**	0.71 (0.14, 1.28)	**0.015**

TC: total cholesterol, BMI: body mass index, UA: uric acid, VAI: visceral adiposity index, SBP: systolic blood pressure, NRI: net reclassification improvement, IDI: integrated discrimination improvement, T2D: type 2 diabetes

^a^ Clinical risk model included age, sex, education, physical activity, smoking status, alcohol intake, waist circumstance, systolic blood pressure, diastolic blood pressure, body mass index, visceral adiposity index, total cholesterol, low-density lipoprotein cholesterol, high-density lipoprotein cholesterol, triglycerides, aspartate aminotransferase, alanine aminotransferase, creatinine, Urea, uric acid, fasting blood glucose, hemoglobin A1c, family history of diabetes, medication use (antihypertensive agents and lipid-lowering agents)

## Discussion

In this prospective cohort study, we found that high variability in visit-to visit TC concentrations, BMI, VAI and SBP were all significantly associated with higher risks of incident T2D during a median of 3.91year follow-up period. As for UA, significant multiplicative interaction was found between variability in UA concentrations and variability in other four metabolic parameters for incident T2D. We also found a graded association between the number of high-variability metabolic parameters and the incidence of T2D. Similar results were found in sensitivity analyses. Furthermore, the addition of variability in metabolic parameters to the clinical risk model which included multiple traditional risk factors, could significantly improve its predictive value for incident T2D.

The association between metabolic parameters and incident T2D among Chinese have been widely analyzed. In Chinese adults, Yu Xu et al. found that each 43 mg/dL increase in TC concentration increases the risk of T2D by 65% (OR: 1.65; 95% CI: 1.47, 1.85) and each 22 mmHg increase in SBP increases the risk of T2D by 47% (OR: 1.47; 95% CI: 1.42, 1.52). Compared to people with BMI < 25.0 kg/m^2^, overweight (BMI: 25.0-29.9 kg/m^2^) and obesity (BMI ≥ 30.0 kg/m^2^) people have a 31% (OR: 1.31; 95% CI: 1.21, 1.41) and 103% (OR: 2.03; 95% CI: 1.78, 2.32) increased risk of developing T2D, respectively [2]. Another study confirmed that compared to individuals with the lowest quartile of VAI, those who had the highest quartile of VAI were at 2.55-fold risk of diabetes (HR: 2.55, 95% CI: 1.58, 4.11) [3]. Besides, Tiange Wang found that compared with the lowest quartile of UA, the highest quartile had an HR for incident diabetes of 2.45 (95% CI: 1.39, 4.33) in men and 1.39 (95% CI: 1.04, 1.84) in women after fully adjustment [4]. However, most previous studies mainly focused on a single metabolic parameter measurement without considering the long-term effect of metabolic parameters changes over time on T2D. To our knowledge, the present study was the first to examine the longitudinal associations of variability in multiple metabolic parameters including visceral adiposity as well as the number of high-variability metabolic parameters with the risk of T2D in a same prospective cohort. We found that high variability in TC concentrations, BMI, VAI and SBP were significantly associated with higher risks of incident T2D. Besides, the risk of incident T2D increased with an increasing number of high-variability metabolic parameters (TC, UA, BMI, VAI and SBP), which suggested that the association of variability in TC, UA, BMI, VAI and SBP with incident T2D were additive. Also, the association we observed were independent of traditional T2D risk factors and thus had incremental value on T2D risk prediction.

The mechanisms underlying the relationship between variability in metabolic parameters and the risk of T2D remain unclear, but several explanations could be suggested. First, many drugs may have unintended effects on lipid levels, BP and other metabolic parameters [13]. Individuals taking multiple medications were more likely to exhibit increased variability parameters, and these individuals may represent a high-risk group. Also, high variability of multiple parameters might be observed in patients with systemic conditions and generalized frailty [14]. Therefore, high metabolic parameters variability may be an epiphenomenon of other diseases that increase T2D risk [15]. Each metabolic parameter will be discussed next. TC concentration variability was reported to be significantly associated with the risk of end-stage renal disease, mortality, myocardial infarction and stroke [15, 16]. A study conducted in Korea found that compared with the lowest decile group, the highest decile group of TC variability showed an increased risk of diabetes development (HR: 1.139; 95% CI: 1.116, 1.163) [17]. In our study, similar results were found and this association may be mediated by endothelial dysfunction [18]. Studies have found that high cholesterol variability was associated with endothelial dysfunction by affecting cerebral blood flow and white matter hyper-intensity load [19, 20]. In addition, the effect of changes in cholesterol levels on insulin secretion was found in both in vivo and in vitro experiments [21, 22]. Results of the association between BMI or SBP variability and metabolic diseases were not identical among different studies [23, 24]. In this study, we found significant association of BMI and SBP variability with incident T2D. As for BMI, its variability could increase oxidative stress and produce low levels of inflammation [25], which has been confirmed to be associated with incident T2D [26]. Also, weight fluctuations can have an impact on decreased immune function [27] and it has confirmed that the immune system plays an important mechanistic role in the development of T2D [28]. As for SBP, on the one hand, BP variability is strongly associated with subclinical inflammation [29]. On the other hand, increased BP variability is a marker of decreasing arterial elasticity and inability to maintain hemodynamic homeostasis [30, 31], which may both have impacts on the development of T2D.

Visceral adiposity also matters. Many studies have confirmed the association between baseline visceral obesity index and various adverse outcomes such as diabetes and hypertension [32, 33], but studies on visceral adiposity variability were limited. An 18-year cohort study found that high VAI trajectory grade was significantly associated with the development of chronic kidney disease (CKD) [34]. Another study that used Chinese visceral adiposity index (CVAI) as the indicator of visceral adipose did not find a significant association between changes in CVAI and carotid plaque risk in a Chinese population [35]. In our study, visit-to-visit variability in VAI was significantly associated with the incidence of T2D. This may be attributed to the correlation between variability in BMI and TC with variability in VAI. The effect of fluctuations in visceral fat on leptin and lipocalin may also furthermore have some effects on the development of insulin resistance and T2D [34], but further studies are still needed to explain this association.

This study has several key strengths. First, this was the first study to investigate the association of metabolic parameters variability with incident T2D and further assess whether they have additive effects on the risk of T2D. Second, as this was a prospective cohort study, the causal relationship could also be demonstrated to some extent. Third, all available confounding factors were adjusted and multiple sensitivity analyses were conducted. However, we also acknowledge several limitations of our study. First, the number of determinations may affect the calculation of the variability. Variability of metabolic parameters in the present study was defined as intraindividual variability of three measurements (2015, 2016, 2017). Second, selection of study population based on the number of health examinations could be subject to selection bias. Furthermore, although researchers would perform OGTT annually, only a small number of the participants were willing to take OGTT examination, which might lead to an underestimation of the incidence of T2D. Finally, although we have adjusted for many potential risk factors for T2D, as we did not collect information on some other proven risk factors for T2D such as sleep duration and sedentary time of participants, we still cannot exclude the possibility that these unmeasured confounders in this study influenced the results. In the future, our findings may need to be further confirmed in large-scale investigation and experimental studies.

## Conclusions

Overall, during a median of 3.91year follow-up, we found that high variability in TC concentrations, BMI, VAI and SBP were significantly associated with higher risks for incident T2D. A graded association between the number of high-variability metabolic parameters (TC, UA, BMI, VAI and SBP) and the incidence of T2D were also observed in this cohort study. Similar results were found in sensitivity analyses. Besides, the addition of variability in different metabolic parameters to the baseline risk model which included traditional risk factors significantly improved its predictive value for incident T2D. Our results provided further evidence for the prevention and control of T2D, which should focus on reducing the variability of metabolic parameters in addition to the average metabolic parameter levels.

## Electronic Supplementary Material

Below is the link to the electronic supplementary material

Supplementary Material 1

## Data Availability

The datasets used and/or analysed during the current study are available from the corresponding author on reasonable request.

## List of Abbreviations

T2D: Type 2 diabetes
BP: Blood pressure
UA: Uric acid
CVD: Cardiovascular disease
TyG: Triglyceride-glucose index
BMI: Body mass index
TC: Total cholesterol
VAI: Visceral adiposity index
SBP: Systolic blood pressure
WC: Waist circumstance
HDL-C: High-density lipoprotein cholesterol
TG: Triglycerides
DBP: Diastolic blood pressure
LDL-C: Low-density lipoprotein cholesterol
AST: Aspartate aminotransferase
ALT: Alanine aminotransferase
FBG: Fasting blood glucose
HbA1c: Hemoglobin A1c
Cr: Creatinine
CV: Coefficient of variation
SD: Standard deviation
VIM: Variability independent of the mean
ARV: Average real variability
OGTT: Oral glucose tolerance test
HR: Hazard ratio
CI: Confidence intervals
NRI: Net reclassification improvement
IDI: Integrated discrimination improvement
CKD: Chronic kidney disease
CVAI: Chinese visceral adiposity index
