# Supplementary material for: Association of variability in metabolic parameters with the incidence of type 2 diabetes: evidence from a functional community cohort
Source: Cardiovasc Diabetol. 2023 Jul 20;22:183. doi: 10.1186/s12933-023-01922-4 (PMC10357611; doi:10.1186/s12933-023-01922-4)
Supplement: Supplementary file 1 — Supplementary Material 1 [file 12933_2023_1922_MOESM1_ESM.docx]

**Additional File 1:**

**Fig. S1** Correlation between variability of different metabolic parameters

**Table S1** Association of number of high-variability parameters or variability score with incident T2D

**Table S2**  Association of incidence of T2D and metabolic parameters variability as a continuous variable (per 5% of CV)

**Table S3** Association of some other parameters variability with the incidence of T2D

**Table S4** Association between incident T2D and the number of high-variability parameters (Sensitivity analyze: measured variability by SD, ARV, VIM)

**Table S5**  Association between number of high-variability of metabolic parameters and incident T2D (Sensitivity analyze: excluding participants experiencing T2D within one year

**Table S6** Association between number of high-variability of metabolic parameters and incident T2D (Sensitivity analyze: adjusted for mean BMI, TC, SBP, UA, VAI)


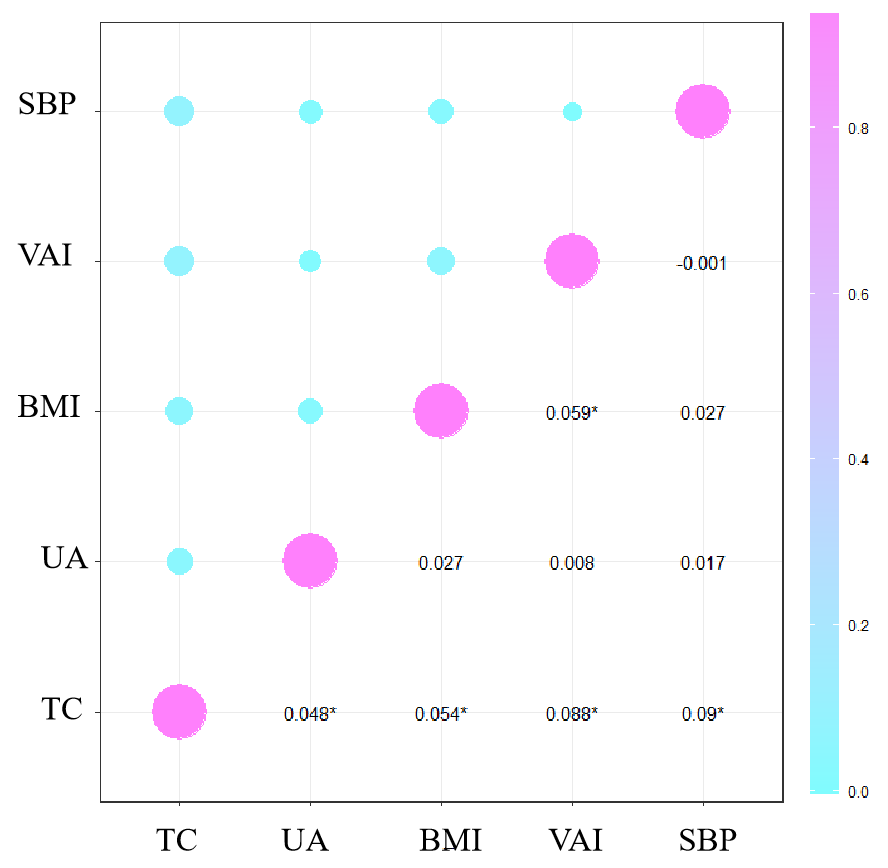


Fig. S1 Correlation between variability of different metabolic parameters

The *number* in the picture indicates the correlation coefficient;

* indicates that the *P* value is less than 0.05, which means that the correlation between the two metabolic parameters is significant.

Table S1 Association of number of high-variability parameters or variability score with incident T2D

|  | Events | Incidence rate | Model 1 ^a^ |  | Model 2 ^b^ |  |
| --- | --- | --- | --- | --- | --- | --- |
|  |  |  | HR (95% CI) | *P* | HR (95% CI) | *P* |
| **Number of high-variability parameters** |  |  |  |  |  |  |
| 0 | 41 | 3.57% |  |  |  |  |
| 1 | 71 | 4.40% | 1.138 (0.774, 1.673) | 0.510 | 1.135 (0.770, 1.673) | 0.522 |
| 2 | 87 | 7.83% | 2.132 (1.469, 3.095) | ＜0.001 | 2.010 (1.378, 2.930) | ＜0.001 |
| 3 | 38 | 8.94% | 2.408 (1.544, 3.756) | ＜0.001 | 2.047 (1.294, 3.239) | 0.002 |
| 4 | 9 | 11.11% | 2.934 (1.423, 6.051) | ＜0.001 | 2.739 (1.302, 5.759) | 0.008 |
| 5 | 3 | 27.27% | 8.169 (2.515, 26.532) | ＜0.001 | 4.473 (1.281, 15.615) | 0.019 |
| *P* for trend | |  |  | ＜0.001 |  | ＜0.001 |

T2D: type 2 diabetes

^a^ Model 1 was adjusted for age, sex, smoking status, alcohol intake, education and physical activity.

^b^ Model 2 was adjusted for age, sex, smoking status, alcohol intake, education and physical activity, waist circumstance, systolic blood pressure, diastolic blood pressure, body mass index, visceral adiposity index, total cholesterol, low-density lipoprotein cholesterol, high-density lipoprotein cholesterol, triglycerides, aspartate aminotransferase, alanine aminotransferase, creatinine, Urea, uric acid, fasting blood glucose, hemoglobin A1c, family history of diabetes, medication use (antihypertensive agents and lipid-lowering agents)

Table S2 Association of incidence of T2D and metabolic parameters variability as a continuous variable (per 5% of CV)

| Metabolic parameters | Model 1 ^a^ |  | Model 2 ^b^ |  |
| --- | --- | --- | --- | --- |
|  | HR (95% CI) | *P* | HR (95% CI) | *P* |
| TC | 1.225 (1.121, 1.338) | < 0.001 | 1.153 (1.054, 1.260) | 0.002 |
| UA | 1.078 (1.983, 1.081) | 0.110 | 1.059 (0.968, 1.159) | 0.213 |
| BMI | 1.632 (1.105, 2.409) | 0.014 | 1.748 (1.177, 2.597) | 0.006 |
| VAI | 1.067 (1.033, 1.102) | < 0.001 | 1.040 (1.004, 1.077) | 0.029 |
| SBP | 1.183 (1.049, 1.333) | 0.006 | 1.167 (1.019, 1.337) | 0.026 |
| ***Interaction effect*** |  |  |  |  |
| UA*TC | 1.047 (1.028, 1.067) | < 0.001 | 1.038 (1.017, 1.059) | < 0.001 |
| UA*BMI | 1.077 (1016, 1.141) | 0.012 | 1.075 (1008, 1.146) | 0.027 |
| UA*VAI | 1.015 (1.007, 1.022) | < 0.001 | 1.009 (1.001, 1.017) | 0.025 |
| UA*SBP | 1.053 (1.021, 1.086) | 0.001 | 1.042 (1.010, 1.075) | 0.010 |

CV: coefficient of variation, TC: total cholesterol, BMI: body mass index, UA: uric acid, VAI: visceral adiposity index, SBP: systolic blood pressure, T2D: type 2 diabetes

^a^ Model 1 was adjusted for age, sex, smoking status, alcohol intake, education and physical activity.

^b^ Model 2 was adjusted for age, sex, smoking status, alcohol intake, education and physical activity, waist circumstance, systolic blood pressure, diastolic blood pressure, body mass index, visceral adiposity index, total cholesterol, low-density lipoprotein cholesterol, high-density lipoprotein cholesterol, triglycerides, aspartate aminotransferase, alanine aminotransferase, creatinine, Urea, uric acid, fasting blood glucose, hemoglobin A1c, family history of diabetes, medication use (antihypertensive agents and lipid-lowering agents)

Table S3 Association of some other parameters variability with the incidence of T2D (per 5% of CV)

| Metabolic parameters | Model 1 ^a^ |  | Model 2 ^b^ |  |
| --- | --- | --- | --- | --- |
|  | HR (95% CI) | *P* | HR (95% CI) | *P* |
| DBP | 1.217 (1.067, 1.387) | 0.003 | 1.161 (1.004, 1.342) | 0.044 |
| WC | 1.075 (0.755, 1.530) | 0.689 | 1.106 (0.768, 1.592) | 0.588 |
| TG | 1.072 (1.030, 1.116) | 0.001 | 1.036 (0.992, 1.082) | 0.109 |
| LDL-C | 1.117 (1.050, 1.189) | < 0.001 | 1.067 (1.000, 1.138) | 0.050 |
| HDL-C | 1.097 (1.012, 1.188) | 0.025 | 1.063 (0.980, 1.153) | 0.138 |
| Cr | 1.159 (1.007, 1.334) | 0.040 | 1.134 (0.980, 1.313) | 0.091 |
| Urea | 1.047 (0.968, 1.132) | 0.251 | 1.052 (0.972, 1.138) | 0.212 |
| ALT | 1.041 (0.996, 1.089) | 0.076 | 1.015 (0.959, 1.075) | 0.607 |
| AST | 1.002 (0.967, 1.038) | 0.913 | 0.987 (0.950, 1.026) | 0.517 |

WC: waist circumstance, DBP: diastolic blood pressure, LDL-C: low-density lipoprotein cholesterol, HDL-C: high-density lipoprotein cholesterol, TG: triglycerides, AST: aspartate aminotransferase, ALT: alanine aminotransferase, Cr: creatinine

^a^ Model 1 was adjusted for age, sex, smoking status, alcohol intake, education and physical activity.

^b^ Model 2 was adjusted for age, sex, smoking status, alcohol intake, education and physical activity, waist circumstance, systolic blood pressure, diastolic blood pressure, body mass index, visceral adiposity index, total cholesterol, low-density lipoprotein cholesterol, high-density lipoprotein cholesterol, triglycerides, aspartate aminotransferase, alanine aminotransferase, creatinine, Urea, uric acid, fasting blood glucose, hemoglobin A1c, family history of diabetes, medication use (antihypertensive agents and lipid-lowering agents)

Table S4 Association between incident T2D and the number of high-variability parameters (Sensitivity analyze: measured variability by SD, ARV, VIM)

| Variability indicators | Group | Events | Incidence rate | Model 1 ^a^ |  | Model 2 ^b^ |  |
| --- | --- | --- | --- | --- | --- | --- | --- |
|  |  |  |  | HR (95% CI) | *P* | HR (95% CI) | *P* |
| **SD** | 1 | 23 | 1.95% |  |  |  |  |
|  | 2 | 160 | 6.00% | 2.653 (1.712, 4.112) | ＜0.001 | 2.239 (1.436, 3.492) | ＜0.001 |
|  | 3 | 41 | 9.65% | 4.198 (2.515, 7.006) | ＜0.001 | 2.674 (1.560, 4.583) | ＜0.001 |
|  | 4 | 25 | 21.19% | 8.739 (4.938, 15.464) | ＜0.001 | 4.786 (2.614, 8.762) | ＜0.001 |
| *P* for trend | | | |  | ＜0.001 |  | ＜0.001 |
| **ARV** | 1 | 34 | 2.87% |  |  |  |  |
|  | 2 | 147 | 5.48% | 1.782 (1.226, 2.389) | 0.002 | 1.525 (1.043, 2.231) | 0.029 |
|  | 3 | 49 | 11.53% | 3.539 (2.281, 5.490) | ＜0.001 | 2.369 (1.496, 3.753) | ＜0.001 |
|  | 4 | 19 | 18.63% | 5.756 (3.271, 10.128) | ＜0.001 | 3.518 (1.939, 6.385) | ＜0.001 |
| *P* for trend | | | |  | ＜0.001 |  | ＜0.001 |
| **VIM** | 1 | 43 | 3.77% |  |  |  |  |
|  | 2 | 155 | 5.70% | 1.40 (1.002, 1.972) | 0.049 | 1.416 (1.007, 1.991) | 0.045 |
|  | 3 | 38 | 8.66% | 2.219 (1.432, 3.439) | ＜0.001 | 1.902 (1.212, 2.982) | 0.005 |
|  | 4 | 13 | 14.29 | 3.559 (1.909, 6.634) | ＜0.001 | 2.593 (1.363, 4.932) | 0.004 |
| *P* for trend | | | |  | ＜0.001 |  | 0.006 |

SD: standard deviation, VIM: variability independent of the mean (VIM), ARV: average real variability, T2D type 2 diabetes

^a^ Model 1 was adjusted for age, sex, smoking status, alcohol intake, education and physical activity.

^b^ Model 2 was adjusted for age, sex, smoking status, alcohol intake, education and physical activity, waist circumstance, systolic blood pressure, diastolic blood pressure, body mass index, visceral adiposity index, total cholesterol, low-density lipoprotein cholesterol, high-density lipoprotein cholesterol, triglycerides, aspartate aminotransferase, alanine aminotransferase, creatinine, Urea, uric acid, fasting blood glucose, hemoglobin A1c, family history of diabetes, medication use (antihypertensive agents and lipid-lowering agents)

Table S5 Association between number of high-variability of metabolic parameters and incident T2D (Sensitivity analyze: excluding participants experiencing T2D within one year

| Variability indicators | Group | Events | Incidence rate | HR (95% CI) | *P* | Variability indicators | Group | Events | Incidence rate | HR (95% CI) | *P* |
| --- | --- | --- | --- | --- | --- | --- | --- | --- | --- | --- | --- |
| **CV** | 1 | 35 | 3.06% | - |  | **SD** | 1 | 19 | 1.62% | - |  |
|  | 2 | 142 | 5.24% | 1.553 (1.067, 2.260) | 0.021 |  | 2 | 143 | 5.40% | 2.446 (1.504, 3.976) | ＜0.001 |
|  | 3 | 33 | 7.88% | 2.070 (1.259, 3.402) | 0.004 |  | 3 | 37 | 8.85% | 2.988 (1.668, 5.353) | ＜0.001 |
|  | 4 | 10 | 11.24% | 2.466 (1.188, 5.119) | 0.015 |  | 4 | 21 | 18.42% | 4.926 (2.541, 9.550) | ＜0.001 |
| *P* for trend | | | | 0.013 | | *P* for trend | | | | < 0.001 | |
| **ARV** | 1 | 29 | 2.46% | - |  | **VIM** | 1 | 37 | 3.26% | - |  |
|  | 2 | 130 | 4.89% | 1.589 (1.054, 2.394) | 0.027 |  | 2 | 138 | 5.11% | 1.444 (1.002, 2.081) | 0.049 |
|  | 3 | 46 | 10.93% | 2.667 (1.636, 4.350) | ＜0.001 |  | 3 | 35 | 8.05% | 2.066 (1.281, 3.331) | 0.003 |
|  | 4 | 15 | 15.31% | 3.357 (1.733, 6.503) | ＜0.001 |  | 4 | 10 | 11.49% | 2.315 (1.122, 4.778) | 0.023 |
| *P* for trend | | | | < 0.001 | | *P* for trend | | | | 0.013 | |

CV: coefficient of variation, SD: standard deviation, VIM: variability independent of the mean, ARV: average real variability, T2D type 2 diabetes

Table S6 Association between number of high-variability of metabolic parameters and incident T2D (Sensitivity analyze: adjusted for mean BMI, TC, SBP, UA, VAI)

| Group | CV |  | SD |  | ARV |  | VIM |  |
| --- | --- | --- | --- | --- | --- | --- | --- | --- |
|  | HR (95% CI) | *P* | HR (95% CI) | *P* | HR (95% CI) | *P* | HR (95% CI) | *P* |
| 1 (Ref.) | - |  | - |  | - |  | - |  |
| 2 | 1.484 (1.048, 2.102) | 0.026 | 2.285 (1.465, 3.564) | ＜0.001 | 1.552 (1.060, 2.271) | 0.024 | 1.430 (1.017, 2.010) | 0.040 |
| 3 | 2.071 (1.306, 3.284) | 0.002 | 2.770 (1.615, 4.479) | ＜0.001 | 2.464 (1.551, 3.915) | ＜0.001 | 1.965 (1.255, 3.078) | 0.003 |
| 4 | 2.535 (1.302, 4.934) | 0.006 | 5.054 (2.756, 9.268) | ＜0.001 | 3.693 (2.026, 6.734) | ＜0.001 | 2.637 (1.388, 5.011) | 0.003 |
| *P* for trend |  | 0.005 |  | ＜0.001 |  | ＜0.001 |  | 0.004 |

CV: coefficient of variation, SD: standard deviation, VIM: variability independent of the mean, ARV: average real variability, T2D type 2 diabetes
